# Improving cartilage phenotype from differentiated pericytes in tunable peptide hydrogels

**DOI:** 10.1038/s41598-017-07255-z

**Published:** 2017-07-31

**Authors:** Enateri V. Alakpa, Vineetha Jayawarna, Karl E. V. Burgess, Christopher C. West, Bruno Péault, Rein V. Ulijn, Matthew J. Dalby

**Affiliations:** 1 Institution for Integrative Medical Biology, Umeå University, SE901 87 Umeå, Sweden; 2 Centre for Cell Engineering, Institute of Molecular, Cell & Systems Biology, College of Medical, Veterinary & Life Sciences, Joseph Black Building, University of Glasgow, Glasgow, G12 8QQ UK; 3 Scottish Polyomics Facility, Wolfson Wohl Cancer Research Centre, College of Medical, Veterinary & Life Sciences, University of Glasgow, Garscube Estate, Glasgow, G61 1QH UK; 4 Centre for Regenerative Medicine and Centre for Cardiovascular Science, University of Edinburgh, Edinburgh, EH16 4UU UK; 5 Orthopaedic Surgery Dept and Broad Stem Cell Research Center, University of California, Los Angeles, USA; 6 Advanced Science Research Center (ASRC), University of New York, New York, NY 10031 USA

## Abstract

Differentiation of stem cells to chondrocytes *in vitro* usually results in a heterogeneous phenotype. This is evident in the often detected over expression of type X collagen which, in hyaline cartilage structure is not characteristic of the mid-zone but of the deep-zone ossifying tissue. Methods to better match cartilage developed *in vitro* to characteristic *in vivo* features are therefore highly desirable in regenerative medicine. This study compares phenotype characteristics between pericytes, obtained from human adipose tissue, differentiated using diphenylalanine/serine (F_2_/S) peptide hydrogels with the more widely used chemical induced method for chondrogenesis. Significantly higher levels of type II collagen were noted when pericytes undergo chondrogenesis in the hydrogel in the absence of induction media. There is also a balanced expression of collagen relative to aggrecan production, a feature which was biased toward collagen production when cells were cultured with induction media. Lastly, metabolic profiles of each system show considerable overlap between both differentiation methods but subtle differences which potentially give rise to their resultant phenotype can be ascertained. The study highlights how material and chemical alterations in the cellular microenvironment have wide ranging effects on resultant tissue type.

## Introduction

Induction of mesenchymal stem cells (MSCs) to undergo chondrogenesis requires the cells to have strong cell-cell interactions and that they maintain a spherical morphology. The added use of growth factors in culture media such as transforming growth factors (TGFs) and bone morphogenetic proteins (BMPs) have also been shown to induce chondrogenesis^1, 2^. However, a common observation when inducing MSCs to form chondrocytes *in vitro* is the expression of type X collagen by the cells^3–6^. Typically, type X collagen is not expressed in the mid-zone of hyaline cartilage and is characteristic of chondrocytes undergoing hypertrophy and endochondral ossification in the deep-zone region of the tissue^7, 8^ suggesting that *in vitro*, the cells differentiate along a mixed phenotypic lineage. Over exposure to compounds such as TGF-β1 is thought to be responsible for increased type X expression^3, 6^ and, as such, biomaterial systems which are able to influence phenotypic expression are highly desirable as a replacement for chemically induced differentiation.

The use of stem cells for engineering cartilage is of particular interest as they facilitate continual development from chondroblasts to terminal differentiation (hypertrophic chondrocytes), a characteristic which is not observed with the use of chondrocytes^9^. Also, induced differentiation of stem cells is able to provide an abundant source of chondrocytes to compensate for the naturally low cell numbers found in cartilage tissue. Cartilage is an avascular tissue type and naturally occuring cell populations are low. To collect and culture mature chondrocytes up to required population numbers is time consuming and costly. For this reason, prefered approaches for engineering cartilage have been to differentiate stem cells *in vitro* which can then be used *in vivo*. Pericytes, or perivascular stem cells, are sourced from the vasculature, inclusive of adipose tissue^10–12^. Thus, they are able to meet the much needed demand of a highly abundant multipotent cell type and are well placed for use in cartilage tissue engineering.

Tuning of material mechanical properties (stiffness) is an effective means of targeting a range of MSC fates, inclusive of chondrogenesis^13–15^. Supramolecular gels have shown enormous potential as model biomaterials to meet this particular challenge^16–19^. There have been major successes in the application of self-assembled peptide based materials as instructive matrices for stem cell growth, where the emphasis has been on the inclusion of biochemical signals, usually comprising matrix protein specific peptidic motifs^18^. There has also been focus on chemical approaches to control gel stiffness, either *via* redesign of building blocks or chemical crosslinking^20^. A number of breakthroughs have shown that stem cells’ growth and differentiation, in addition to biochemical signals, are highly sensitive to physical stimuli presented by their immediate environment^21, 22^. Specifically, mechanical^21^ (i.e. gel stiffness) and structural/topographical factors^23^ of the cell-contacting matrix play crucial roles that have, in some cases, been shown to be more powerful than soluble biochemical signals^24^.

Previously, we had shown that pericytes cultured in supramolecular peptide hydrogels were able to undergo differentiation into a number of cell lineages when the hydrogels were tuned to various stiffnesses^25^. In this study, an interesting find was the differentiation of pericytes along the chondrogenic lineage when cells were cultured in 13 kPa hydrogel, contrary to the previously observed myogenic development in other biomaterials with similar stiffnesses^24, 26^. The distinction of which can be explained by the use of a nanofiber structured hydrogel which the cells interact differently with compared to crosslinked materials^25^.

As this was an unusual observation for cellular differentiation in mechanically tuned substrates, where cell behaviour is contradictory to the norm, this study aimed to ascertain the properties of chondrocytes that develop in the Fmoc-F_2_/S hydrogels. We do this by further investigating the chondrogenic induction of pericytes, if Fmoc-F_2_/S hydrogels are able to sustain development in the longer term and whether the effective cellular development can be enhanced with the aid of chondrogenic induction media.

## Results

### Fmoc-F_2_/S hydrogels act as biomaterial substrate to promote chondrogenesis of pericytes

**Figure 1:**
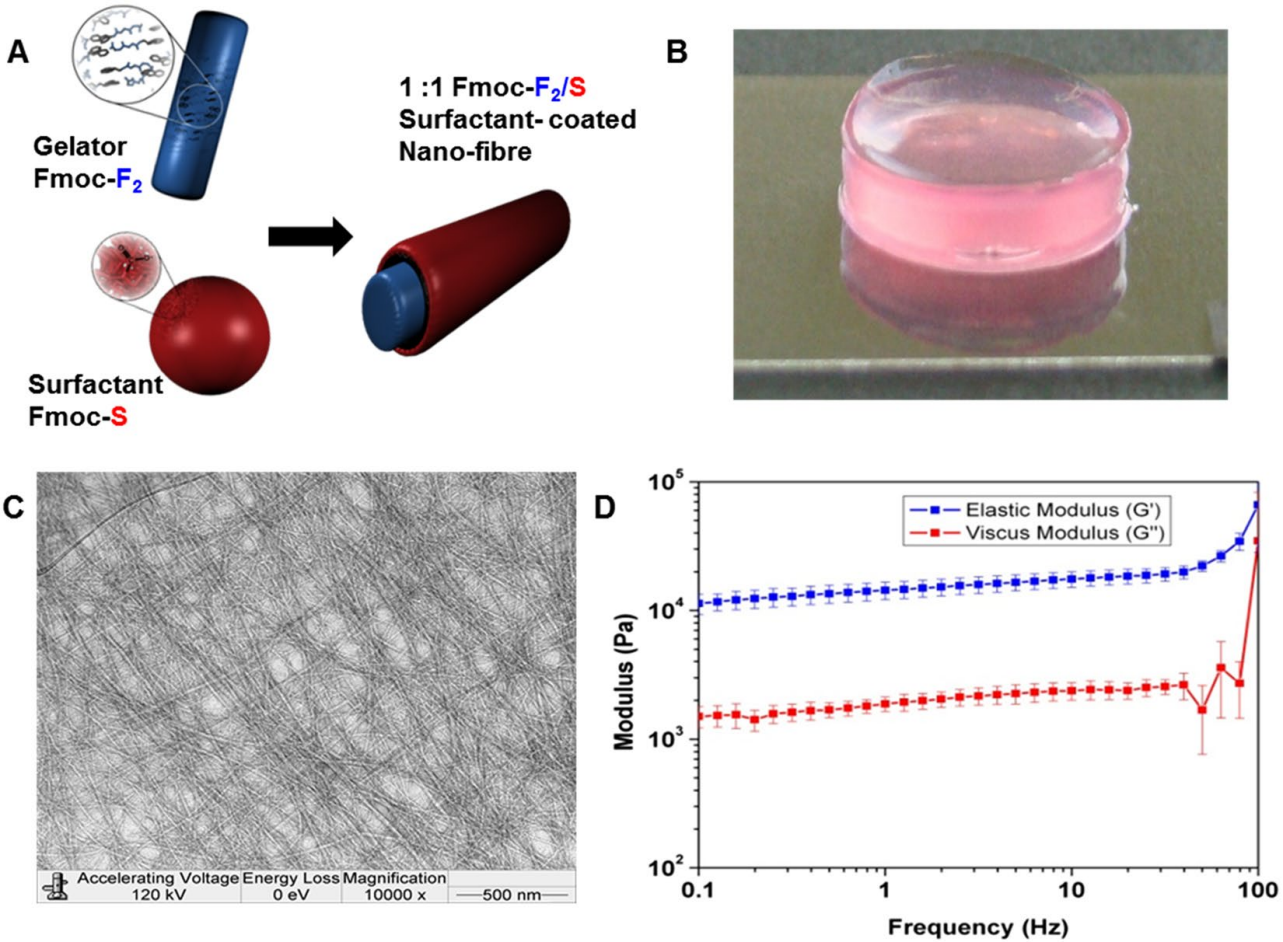
Self-assembly of two-component gelators. (**A**) Schematic presentations of the building blocks, gelator Fmoc-F_2_, surfactant Fmoc-S and surfactant coated nano fiber Fmoc-F_2_/S. (**B**) Macroscopic image for gel in culture media. (**C**) TEM image of hydrogel showing fibrous morphology. (**D**) Oscillatory rheology of the gels showing elastic moduli of 15.5 kPa.

We recently reported on the use of co-assembled hydrogels of the well-known gelator fluorenylmethoxycarbonyl (Fmoc)-diphenylalanine (F_2_)^27, 28^ and surfactant-like Fmoc-serine (Fmoc-S) to produce cyto-compatible core/shell nanofibers that may be crosslinked upon exposure to cell culture media, resulting in gelation (Fig. 1A and B)^29^. The mechanical properties of the Fmoc-F_2_/S hydrogels were tuned by careful control of the peptide concentration in the pre-gel liquid before initiating cross linking with introduction to culture media, allowing gelation to occur as published previously^25^. The supramolecular hydrogels were therefore created using peptide concentrations that allow the formation of gels with moduli similar to that reported for chondrons^30, 31^. Oscillatory rheology of the hydrogel shows that the gels possess elastic moduli of 15.5 kPa (Fig. 1D). The G′, elastic modulus, exceeds the viscous modulus G″, signifying that the hydrogel is an elastic material. The nanoscale features and hydrophilic chemistry presented by Fmoc-S on the surface allows nanoscale hydrogel fibres to adsorb proteins which enable indirect contact with cell surface receptors facilitating the cell-material interaction needed to interpret biomaterial qualities (Fig. 1C).

**Figure 2:**
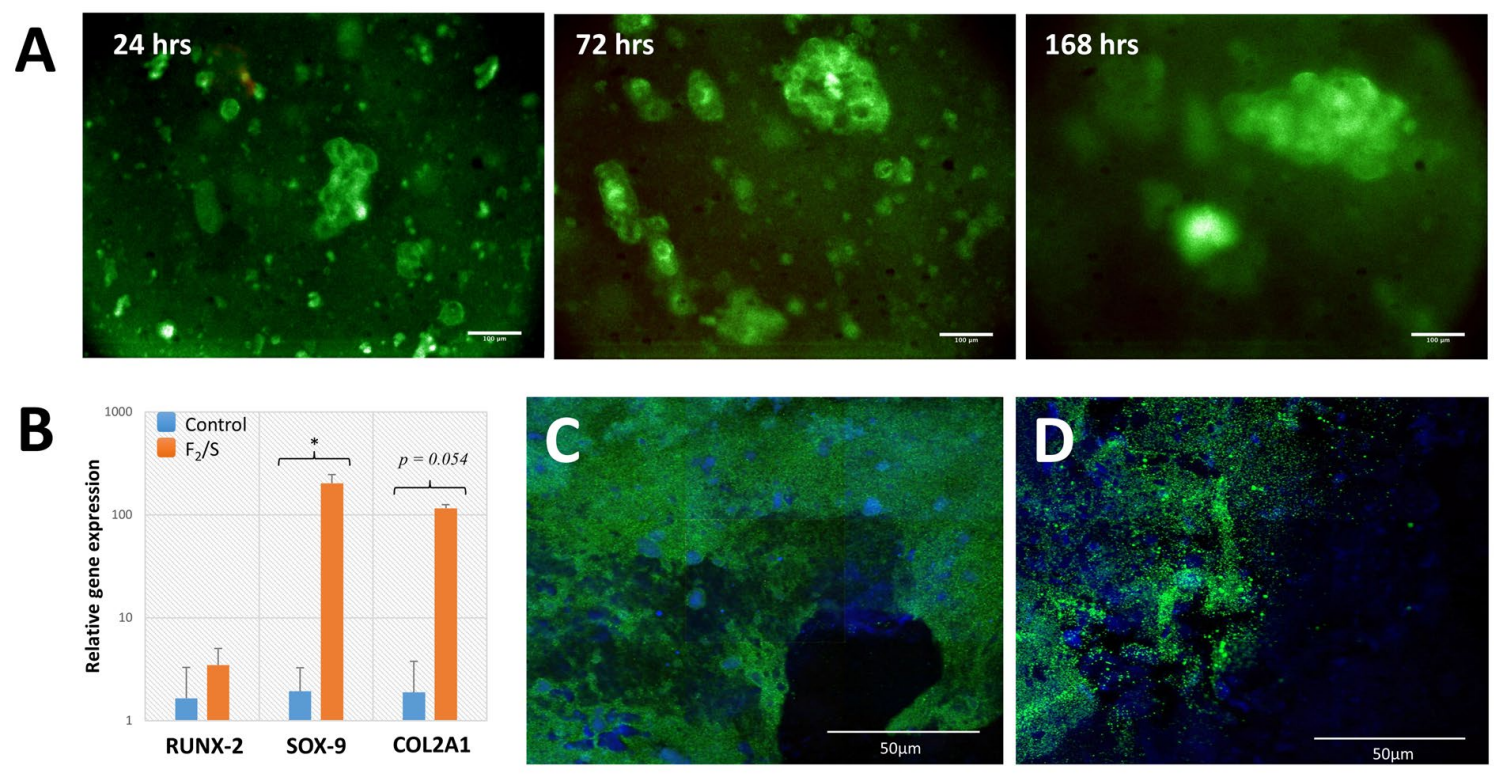
(**A**) Human adipose derived pericyte cultured within Fmoc-F_2_/S hydrogels. Cells were encapsulated in F_2_/S hydrogels and maintained in unsupplemented basal media for up to 1 week. Cells were checked for viability by fluorescence detection of Syto 10 (green) for live cells and ethidium homodimer-1 (red) for dead cells after 1, 3 and 7 days. (**B**) QRT-PCR analysis for gene expression of pericyte cells cultured within 15.5 kPa Fmoc-F_2_/S hydrogels. Cells were assessed for the production of chondrogenic biomarkers RUNX-2, SOX-9 & type II collagen (COL2A1) after one week in culture. (**C** & **D**) Confocal microscopy images of immunofluorescently stained F_2_/S hydrogels cultured with pericytes for 28 days. Pericytes were checked for chondrogenic development by staining for aggrecan production (**C**) and type II collagen (**D**) both ascertained through green fluorescence. Cell populations are indicated by staining the cell nucleus with DAPI (blue). The images are mosaics of a 3 × 3 tile scan, each acquired from random positions of the hydrogel. Scale bar in **A** is 100 µm, in **C** & **D** is 50 µm. In B, Error bars denote the standard error where p < 0.05 as calculated using unpaired student t-test.

Promotion of chondrogenic development *in vitro* generally requires that cells are cultured within a three dimensional construct in order to maintain a typical rounded morphology and eliminating the dedifferentiation effects of monolayer culture^32–34^. The formation of aggregates in culture is of particular advantage as it has been shown to promote chondrogenic development in stem cells^35, 36^. Pericytes cultured within the 15.5 kPa Fmoc-F_2_/S hydrogels were observed to have good viability with cells forming small clusters (aggregates) over time (Fig. 2A). Pericyte differentiation was assessed by monitoring gene expression levels of RUNX-2, SOX-9 and type II collagen after 1 week which showed increased expression levels of all three genes compared to the negative control with SOX-9 and type II collagen in particular showing a statistically significant increase (Fig. 2B).

Cells were then cultured over a longer term (5 weeks) within the Fmoc-F_2_/S substrate and were subsequently immunofluorescently stained for type II collagen and aggrecan production. Confocal microscopy imaging demonstrated the presence of both proteins indicating successful differentiation of the pericytes into chondrocytes (Fig. 2C and D).

### Fmoc-F_2_/S hydrogel promotes lessens formation of type X collagen while balancing aggrecan and type II collagen ratios

Phenotypic characteristics of differentiated pericytes were monitored by focusing on the relative expression levels of type II collagen, aggrecan and type X collagen after long-term culture (35 days). Comparisons were carried out using pericytes cultured in the 15.5 kPa Fmoc-F_2_/S gels in the presence (+) and absence (−) of chondrogenic induction medium. In addition, pericytes were also cultured in alginate hydrogels with chondrogenic induction medium in order to make a comparison with a conventionally used hydrogel system for chondrogenesis that is also used in MACI^37–41^. It is noteworthy to mention that many studies which use alginate for chondrogenesis of MSCs, culture the cells using low serum (1% FBS) concentrations. As chondrogenesis was originally observed in Fmoc-F_2_/S substrates using standard basal media composition (10% FBS), this study retains the conditions in which the original observation was made for both alginate and Fmoc-F_2_/S.

**Figure 3:**
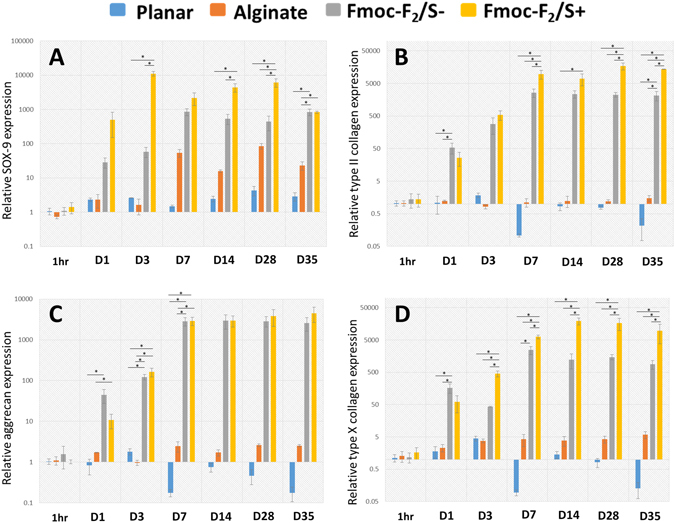
Quantitative PCR analysis assessing chondrocyte development of pericytes encapsulated in Fmoc-F_2_/S hydrogels with (+), without (−) chondrogenic induction and in alginate hydrogels (ALG) also cultured with chondrogenic induction media. Cells were assessed for gene expression of the cartilage biomarkers SOX-9 (**A**), type II collagen (**B**), aggrecan (**C**) and type X collagen (**D**) up to 35 days in culture. Expression levels of all four biomarker were observed as elevated for all culture systems with the highest expression levels noted for pericytes cultured in Fmoc-F_2_/S hydrogels. Gene expression was compared against pericytes cultured on glass cover slips (undifferentiated on planar substrate) as a negative control. Error bars are standard error of the mean; * indicate significant difference between groups as determined by one-way ANOVA followed by Bonferroni post hoc test where p < 0.05; n = 4.

**Figure 4:**
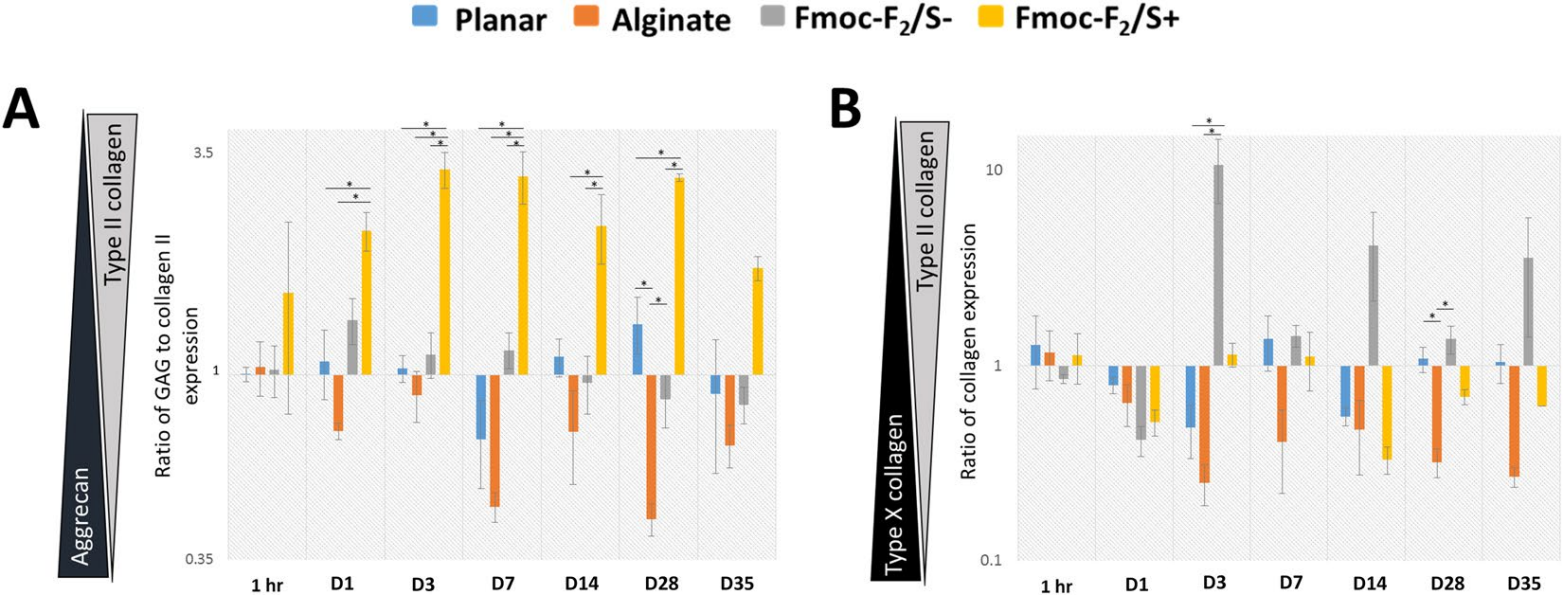
Comparison of chondrocyte expression of pericyte cells differentiated over a 5 week period using 15.5 kPa Fmoc-F_2_/S hydrogels in the presence (+) and absence (−) of chondrogenic induction media. Cells were also differentiated using alginate hydrogels and chondrogenic induction media. (**A**) Expression of type II collagen relative to aggrecan showed higher type II collagen transcription for cells cultured in Fmoc-F_2_/S + hydrogels. (**B**) Expression of type II collagen relative to type X collagen. Gene expression was compared against pericytes cultured on glass cover slips (undifferentiated on planar substrate) as a negative control. Error bars are standard error of the mean; * indicate significant difference between groups as determined by one-way ANOVA followed by Bonferroni post hoc test where p < 0.05; n = 4.

Gene expression profiles of pericytes showed an increase in all cartilage biomarkers over 1 week after which trends held steady up to 35 days indicating differentiation of pericytes. Marker expression levels from pericytes in both Fmoc-F_2_/S+ and Fmoc-F_2_/S− hydrogels were distinctly higher than those in alginate. The Fmoc-F_2_/S+ showed the highest production of SOX-9, type II collagen and type X collagen respectively (Fig. 3). Assessment of collagen and glycosaminoglycan production relative to each other showed that the outcome of this was dependent on which culture system was used. Type II collagen production was highest in Fmoc-F_2_/S+, having on average 2.6 fold higher concentrations compared to aggrecan (Fig. 4A). Aggrecan content was largest when alginate was used (Fig. 4A). Pericytes cultured in Fmoc-F_2_/S− hydrogels exhibited an overall balance between type II collagen and aggrecan expression (0.89x, Fig. 4A).

Over expression of type X collagen compared to type II is known to be prevalent in most *in vitro* systems that differentiate stem cells into chondrocytes. This phenomenon is thought to be due to the culture system having an over exposure to transforming growth factor β1 (TGF-β1)^3, 6^ and also because of the use of ascorbic acid^42–44^, both of which are typically used in formulating chondrogenic induction medium. While Fmoc-F_2_/S+ and alginate produced the collagen II/collagen X ratio with greater collagen X weighting; it was seen, however, that F_2_/S−, which is absent of the tailored medium, produced the desired ratio with greater collagen II weighting (Fig. 4B), highlighting the influence of the supplemented medium on resultant cellular differentiation.

### Chondrocyte metabolome highlights pathways that are causal of phenotypic differences

**Figure 5:**
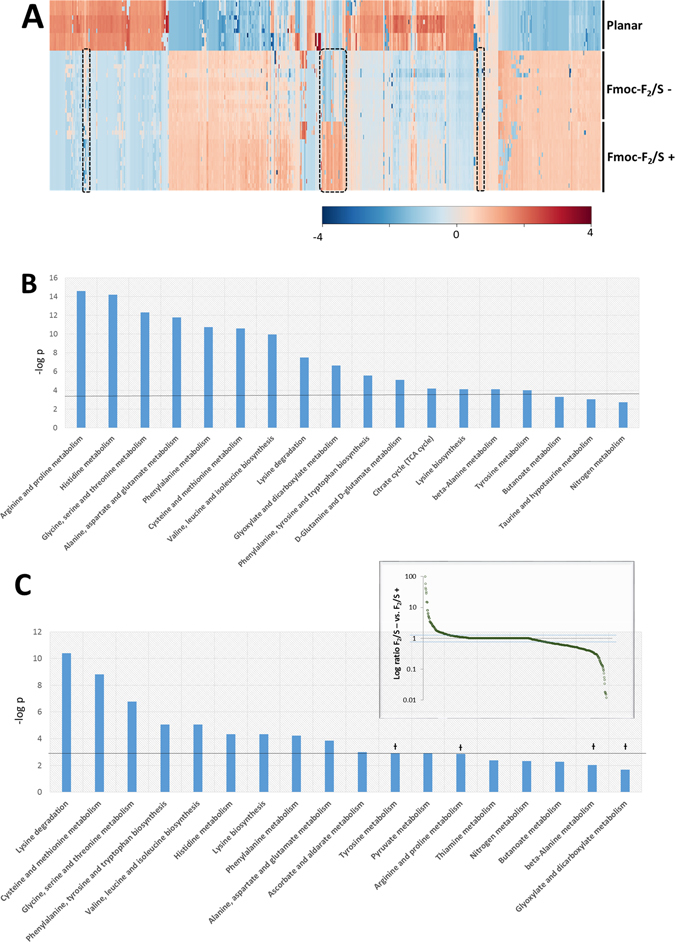
Analysis of metabolite masses detected from pericyte cell extracts undergoing chondrogenesis (n ≥ 12). (**A**) Hierarchical cluster analysis of metabolic MS masses detected using LC-MS for pericytes cultured on planar substrates (undifferentiated) and cultured in Fmoc-F_2_/S in the presence or absence of chondrogenic induction media 35 days. (**B**) Metabolites were mapped to metabolic pathways to ascertain which cell processes are significantly changed from the control. (**C**) Comparisons between Fmoc-F_2_/S− and Fmoc-F_2_/S+ isolate metabolite masses that differ between the two by more than two fold (inset) giving insight into processes that lead to the altered chondrocyte phenotype between Fmoc-F_2_/S− and Fmoc-F_2_/S+ Pathways marked with Ɨ were observed to be significantly different from undifferentiated cells but not between Fmoc-F_2_/S− and Fmoc-F_2_/S+ suggesting influence during chondrogenesis but do not contribute to the observed differences in chondrocyte phenotype.

Metabolomics data were generated using high-resolution LC-MS analysis of denatured cell extracts of pericytes cultured in the Fmoc-F_2_/S+/− hydrogel systems. Comparisons between these two systems allowed identification of cell processes that are affected by the presence of induction medium and which subsequently lead to differences in phenotypic expression. A generalised overview of the metabolome using hierarchical cluster analysis showed that, on the whole, the metabolite profiles of Fmoc-F_2_/S+/− systems, although distinct from the undifferentiated control set, were similar to each other with slightly higher metabolite abundances detected on the Fmoc-F_2_/S+ substrate (Fig. 5A). Detected metabolite masses were mapped to known pathways to ascertain which areas of metabolism were most differentiated from the control set. The pathways showing the most significant change were those involved in amino acid metabolism and energy generating processes such as the TCA cycle (Fig. 5B). Of these, the most changed significantly were metabolites involved in arginine and proline metabolism. A pathway which contributed to the development of collagen and the synthesis of polyamines. Polyamines are known to play an important role in proliferation and differentiation^45, 46^, and in particular, development of chondrocytes^47^.

Closer inspection between Fmoc-F_2_/S+ and Fmoc-F_2_/S− reveal pockets of distinction between the two. These potentially highlight metabolic processes that ultimately result in the phenotypic differences observed in Fig. 4.Sample sets were compared against each other and a nominal threshold of 2 was set to isolate metabolites considered as distinct from one another. From the total population (734), 23.4% of these were considered to be of interest. When mapped to metabolic processes, the pathways that had the greatest number of hits and therefore most contrasting turnover were centred mainly in amino acid metabolism. Observed differences between Fmoc-F_2_/S− and Fmoc-F_2_/S+ therefore indicate differences in the protein make up between both systems. Pathways such as tyrosine metabolism and arginine & proline metabolism, however, showed no significant change between both systems suggesting that they are requisite for chondrocyte development but do not necessarily play a role in the observed shifts in phenotype between Fmoc-F_2_/S− and Fmoc−F_2_/S+ (Fig. 5C).

Significantly changed pathways involved in energy generation, such as the TCA cycle, although different from undifferentiated cells showed no discernible difference between Fmoc-F_2_/S- and Fmoc-F_2_/S+ as cells in both substrates are considered to be similarly active.

## Discussion

The use of the biomaterial alone not only instigates pericytes to undergo chondrogenesis but the cells are also able to sustain continual development as observed through the constant production of the chondrogenic markers over a longer time in culture. The use of induction medium with the hydrogels causes a phenotypical imbalance, most notably with the production of collagen. No difference between the two systems (with and without induction media) are noted with regards to aggrecan production, suggesting that induction medium is better tailored to collagen development.

The type of collagen formed, however, is affected by the induction media as generally higher type X collagen content is observed in these systems over type II collagen. This correlates with the gene expression profiles observed in Fig. 2 where initial chondrogenesis of pericytes is assessed. In the absence of the induction media, RUNX-2 expression levels is lowered compared to SOX-9. Subsequently, formation of type X collagen, which is pre-empted by RUNX-2, is lowered and we observe higher type II collagen content. Relative levels of the gene markers in alginate were significantly lower than the Fmoc-F_2_/S hydrogels which may be due to the difference in the cell type used, as opposed to MSCs for example, as well as the deviation from the typical use of low serum concentrations. Notwithstanding, comparative production of chondrogenic markers in alginate show that the use of induction media for differentiation also gives a higher proportion of type X collagen formation over type II collagen. Levels that are comparable with Fmoc-F_2_/S+ (Fig. 4B). Typically observed *in vitro*, type X collagen imbalance suggests greater population of hypertrophic chondrocytes^5, 6, 48^, thought to occur due to the initiation of some osteogenic activity^3, 6^. The detection of type X collagen in the Fmoc-F_2_/S- system however, indicates that its production is not completely eliminated but significantly reduced when chemical induction is avoided.

The interplay between collagen and glycosaminoglycan content is noteworthy as the distribution of one relative to the other in native cartilage plays an important role in the functional output of the tissue as a whole. The zonal structure of native hyaline cartilage, shows increased abundance in glycosaminoglycan content moving from the articular surface through the superficial, mid and deep zones where there is the greatest resistance to compressive loading^8, 49, 50^. Therefore, in order to impart better functionality when healing damaged cartilage tissue, the implication of balancing the abundance of type II collagen and aggrecan when developing cartilage tissue *in vitro* through mechanical and chemical substrate design is of particular importance, as it should best match the required structure of the lost tissue. The balance achieved with using Fmoc-F_2_/S− (Fig. 4A) allows for potential formation of a tissue type that is not overly inflexible due to higher collagen content than is observed when including induction medium.

While the findings from the metabolomics study are preliminary, they demonstrate correlation of cell behaviour with their microenvironment and a means of acutely discriminating between potential sub-phenotypes of a particular cell type. That is, distinguishing between finer details that cannot be simply reflected in ‘positive’ detection of specific markers. This however requires further experimental design and implementation beyond the scope of this study.

## Methods

### Formation of the Fmoc hydrogels

Pre gelation mixture was prepared by mixing diphenylalanine (F_2_) and serine (S) powders (both capped at the N-terminal with fluorenylmethoxycarbonyl (Fmoc)) in 14 mL glass vials and suspending the powders to a 30 mM peptide concentration in sterile/distilled H_2_O. 0.5 M NaOH was added dropwise until the powders were fully dissolved. The vial was mixed with alternated vortexing and sonication and then 0.5 M HCl was added until the desired pH was reached (7.5–8.0). Prior to use, Fmoc-F_2_/S pre gelation mixtures were sterilized under UV light for 45 min.

### Alginate solution

A 1.2% (w/v) alginate solution was made by dissolving 0.360 g of alginate powder (Sigma) slowly in 30 ml of phosphate buffered saline containing a magnetic stirrer. The solution was then autoclaved at 120 °C for 20 minutes.

### Rheology

To assess the mechanical properties of the hydrogels, dynamic frequency sweep experiments were carried out on a strain-controlled rheometer (Kinexus rotational rheometer from Malvern) using a parallel-plate geometry (20 mm) with a 0.50 mm gap. An integrated temperature controller was used to maintain the temperature of the sample stage at 25 °C. Precautions were taken to minimize solvent evaporation and to keep the sample hydrated: a solvent trap was used and the atmosphere within was kept saturated. To ensure the measurements were made in the linear viscoelastic regime, an amplitude sweep was performed and the results showed no variation in elastic modulus (G′) and viscous modulus (G″) up to a strain of 1%. The dynamic modulus of the hydrogel was measured as a frequency function, where the frequency sweeps were carried out between 1 and 100 Hz. The measurements were repeated at least three times to ensure reproducibility.

### Transmission electron microscopy (TEM)

Carbon-coated copper grids (No. 400) were glow discharged for 5 s and placed shiny side down on the surface of the hydrogel for less than 5 s. Excess sample was removed by blotting with a filter paper and then 10 mL of negative stain (Nanovan: 1% aqueous methylamine vanadate, obtained from Nanoprobes) was placed on the top of the sample on the grid and allowed to dry for 10 mins. The dried specimens were then imaged using a LEO 912 energy filtering transmission electron microscope operating at 120 kV fitted with a 14 bit/2 K Proscan CCD camera. Fiber diameters were measured using ImageJ software version v1.43 u.

### Pericyte isolation

Adipose tissue was collected with prior informed and written consent from healthy adult volunteers (n = 3) undergoing cosmetic lipectomy procedures. Permission for tissue collection and subsequent experimental protocols were granted and carried out in accordance with stated guidelines by the South East Scotland Research Ethics Committees (SESREC 10/S1103/45) in Edinburgh.

Pericytes were isolated from adipose tissue from adult donors undergoing cosmetic liposuction by Flow Activated Cell Sorting (FACS) using a FACS Aria II (BD Biosciences) based on our established protocols^10^ and is summarised below.

Adipose tissue was enzymatically digested with type II collagenase (1 mg/ml, Sigma-Aldrich) for 30 mins in a shaking waterbath at 37 °C to obtain the Stromal Vascular Fraction (SVF). SVF was then stained with the following antibodies; CD146-Alexa647 (1:100, AbD Serotec, Raleigh, NC), CD45 APC-cy7, CD31-FITC, and CD34-PE (1:100, all from BD Biosciences, San Jose, CA). Pericytes were sorted to homogeneity based on the following phenotype CD146+, CD45−, CD34− and CD31−. Immediately following FACS, pericytes were seeded onto 0.1% gelatin coated wells at a density of 20,000 cells/cm^2^ in EGM-2 media (Lonza) in a humidified incubator with 5% CO_2_ at 37 °C. When confluent, cells were detached from the cultureware using 0.25% trypsin and split at a ratio of 1:6 and cultured in DMEM + 20% FCS for all subsequent passages. Media was changed 3 times per week. Purity of pericyte cultures was confirmed by flow cytometry (Supplementary data, Figure S1).

### Cell Culture and Reagents

Pericytes were tripsinised from the culture well flasks, pelleted by centrifugation and resuspended in either the Fmoc-F_2_/S or the alginate solution.

Cell laden alginate beads were made by pipetting 300 µL of the alginate solution into a 100 mM calcium chloride solution. They were allowed to cure at room temperature for 5 minutes before removing the calcium chloride solution. Hydrogels were then washed twice with PBS solution and 500 μl of culture media added to each well.

300 µL of the Fmoc-F_2_/S cells suspension was dispensed into 24 well culture plates containing 500 μl of culture media. Culture plates were incubated under humidified atmosphere of 5% CO_2_ at 37 °C for approximately 1 hr to allow the Fmoc-F_2_/S hydrogels to fully cure. Following this, the media in both the peptide and alginate hydrogels were changed every 24 hours in the first two days of preparation and every twice weekly after that.

Chondrogenic differentiation in one subset of the Fmoc-F_2_/S and in alginate was induced using DMEM containing 10% FBS, insulin (6.25 μg/ml), dexamethasone (10 nM), ascorbate-2-phosphate (50 nM), transforming growth factor (TGF-β1, 10 ng/ml) and sodium pyruvate (110 μg/ml).

### Cell viability

Culture media surrounding the biomaterials were aspirated to waste and washed once with warm PBS solution. A working solution containing both Syto 10 and ethidium homodimer-2 dyes was made in PBS (1:500 v/v). 500 µL of the dye solution was added to the hydrogels and the samples incubated in the dark at room temperature for 15 minutes.

Following this, the hydrogels were then fixed at room temperature with 4% formaldehyde solution for at least 15 minutes before viewing under a microscope.

### Immunofluorescence cell staining

After 28 days in culture (unless otherwise stated), the cells were washed once in PBS and fixed with 10% formaldehyde at 37 °C for 15 min. When fixed, the samples were permeabilised using a buffer solution (10.3 g sucrose, 0.292 g NaCl, 0.06 g MgCl_2_, 0.476 g Hepes buffer, 0.5 ml Triton X, in 100 ml water, pH 7.2) at 4 °C for 5 min. The samples were then incubated at 37 °C for 5 min in 1% BSA/PBS, followed by the addition of the primary antibody (1:50 in 1% BSA/PBS, monoclonal anti-human collagen type II and aggrecan raised in mouse (IgG1), Santa Cruz Biotechnology Inc) for 1 h (37 °C). The samples were then washed in 0.5% Tween 20/PBS (5 min, ×3). A secondary, biotin-conjugated antibody (1:50 in 1% BSA/PBS, monoclonal anti-mouse (IgG), Vector Laboratories, Peterborough, UK) was added for 1 h (37 °C) followed by washing. A FITC conjugated streptavidin third layer was added (1:50 in 1% BSA/ PBS, Vector Laboratories, Peterborough, UK) at 4 °C for 30 min, and given a final wash.

### QRT-PCR analysis

RNA extractions from cells cultured on hydrogel biomaterials were done using the Trizol extraction reagent (Invitrogen). Cells cultured for on culture well plastic had RNA retrieved using RNeasy micro kit (Qiagen), both protocols were carried out as per manufacturer’s instructions. Reverse transcription to obtain cDNA was done using Quantitech reverse transcription kit (Qiagen) for all samples, also according to the manufacturer’s protocol. Cells were cultured and harvested at time points 1hr, 1, 3, 7, 14, 28 and 35 days in culture.

Amplification by qRT-PCR was done using human specific primers (Eurofins MWG Operon) detailed in Table 1. PCR was carried out using a 7500 Real time PCR system & software (Applied Biosystems). Samples had a total reaction volume of 20 µL containing 2 µL of diluted cDNA, each reverse and forward primer at a final concentration of 100 µM and analysed using SYBR green chemistry (Qiagen). For PCR amplification samples were held at 50 °C for 2 minutes then 95 °C for 10 min then amplified using 95 °C for 15 s and 60 °C for 1 min for 40 cycles. The specificity of the PCR amplification was checked with a heat dissociation curve (measured between 60–95 °C) done subsequent to the final PCR cycle. Gene expression levels were standardised using GAPDH as an internal control. Quantification analysis was performed using the comparative ΔΔCt method^51^ and gene expression expressed as fold change relative to the control sample.

**Table 1:** Real time PCR primers used to quantify mRNA expression from human genes.

Gene		
RUNX-2	Forward	5′-GGT CAG ATG CAG GCG GCC-3′
	Reverse	5′-TAC GTG TGG TAG CGC GTC-3′
SOX-9	Forward	5′-AGA CAG CCC CCT ATC GAC TT-3′
	Reverse	5′-CGG CAG GTA CTG GTC AAA CT-3′
Aggrecan (ACAN)	Forward	5′-TAC ACT GGC GAG CAC TGT AAC-3′
	Reverse	5′-CAG TGG CCC TGG TAC TTG TT-3′
Collagen type II (COL2A1)	Forward	5′-GTG AAC CTG GTG TCT CTG GTC-3′
	Reverse	5′-TTT CCA GGT TTT CCA GCT TC-3′
Collagen type X (COL10A1)	Forward	5′-CAC CTT CTG CAC TGC TCA TC-3′
	Reverse	5′-GGC AGC ATA TTC TCA GAT GGA-3′
GAPDH	Forward	5′-ACC CAG AAG ACT GTG GAT GG-3′
	Reverse	5′-TTC TAG ACG GCA GGT CAG GT-3′

Samples were assayed in quadruplicate and gene expression was expressed as mean ± SEM.

### Metabolomic analysis

Metabolite extraction from cells cultured on hydrogels and control samples for 1 week was done using ice cold chloroform:methanol:water (1:3:1,v/v) on a shaker for 1 h maintained at 4 °C. Samples were centrifuged and 10 μL of the supernatant injected on to the LC-MS system.

The LC separation was carried out using hydrophilic interaction chromatography with a ZIC-HILIC 150 mm × 4.6 mm, 5 μm column (Merck Sequant), operated by an UltiMate liquid chromatography system (Dionex, Camberley, Surrey). The LC mobile phase was run with 0.1% formic acid in water (A) and 0.08% formic acid in acetonitrile (B). The mobile phase was run at a linear gradient for 30 minutes from 20–80% A, maintained at 5% A for 10 minutes and then re-equilibrated to 20% A. Mass spectrometric detection was performed using an Orbitrap Exactive (Thermo Fisher Scientific, Hemel Hempstead, U.K.) within the mass range m/z 70–1400 in polarity switching mode.

Chromatographic peak selection and metabolite identification were done using Ideom/MzMatch excel interface^52, 53^ and measured peak intensities by LC-MS were normalised against protein content as measured using the Bradford assay as detailed previously^54^. Metabolite identification was done using a set of known standards to define mass and chromatographic retention times. Putative metabolites were also identified on this basis using predicted retention times as described by Creek *et al*.^55^.

### Statistical Analysis

Analysis of variance (ANOVA) and Bonferroni post hoc tests were performed using GraphPad prism software to compare more than two study groups. Statistical significance is noted where the calculated p value is less than 0.05 using four biological replicates unless otherwise stated.

Multivariate analysis of the LC-MS data and metabolite pathway mapping were done using Metaboanalyst 2.0^56^.

### Data availability

Raw data generated from this study is available from http://dx.doi.org/10.5525/gla.researchdata.344.

## Electronic supplementary material

Supplementary information

## References

[1] Muraglia A (2003). Formation of a chondro-osseous rudiment in micromass cultures of human bone-marrow stromal cells. Journal of Cell Science.

[2] Sekiya I, Vuoristo JT, Larson BL, Prockop DJ (2002). *In vitro* cartilage formation by human adult stem cells from bone marrow stroma defines the sequence of cellular and molecular events during chondrogenesis. Proceedings of the National Academy of Sciences of the United States of America.

[3] Cooke ME (2011). Structured three-dimensional co-culture of mesenchymal stem cells with chondrocytes promotes chondrogenic differentiation without hypertrophy. Osteoarthritis and Cartilage.

[4] Cui X, Hasegawa A, Lotz M, D’Lima D (2012). Structured three-dimensional co-culture of mesenchymal stem cells with meniscus cells promotes meniscal phenotype without hypertrophy. Biotechnology and Bioengineering.

[5] Karlsson C (2007). Differentiation of human mesenchymal stem cells and articular chondrocytes: Analysis of chondrogenic potential and expression pattern of differentiation-related transcription factors. Journal of Orthopaedic Research.

[6] Perrier E (2011). Analysis of collagen expression during chondrogenic induction of human bone marrow mesenchymal stem cells. Biotechnology Letters.

[7] Hunziker EB, Michel M, Studer D (1997). Ultrastructure of adult human articular cartilage matrix after cryotechnical processing. Microscopy Research and Technique.

[8] Responte, D. J., Natoli, R. M. & Athanasiou, K. A. Collagens of Articular Cartilage: Structure, Function, and Importance in Tissue Engineering. *Critical Reviews in Biomedical Engineering* **35** (2007). 10.1615/critrevbiomedeng.v35.i5.20 19392643

[9] Vonschroeder HP, Kwan M, Amiel D, Coutts RD (1991). The use of polylactic acid matrix and periosteal grafts for the reconstruction of rabbit knee articular defects. Journal of Biomedical Materials Research.

[10] Corselli M (2013). Identification of perivascular mesenchymal stromal/stem cells by flow cytometry. Cytometry. Part A: the journal of the International Society for Analytical Cytology.

[11] Crisan M (2008). A perivascular origin for mesenchymal stem cells in multiple human organs. Cell Stem Cell.

[12] West, C. C. *et al*. Prospective purification of perivascular presumptive mesenchymal stem cells from human adipose tissue: process optimization and cell population metrics across a large cohort of diverse demographics. *Stem Cell Research & Therapy* **7**, doi:10.1186/s13287-016-0302-7 (2016). 10.1186/s13287-016-0302-7 PMC4815276 27029948

[13] Allen JL, Cooke ME, Alliston T (2012). ECM stiffness primes the TGF beta pathway to promote chondrocyte differentiation. Molecular Biology of the Cell.

[14] Discher, D. E., Janmey, P. & Wang, Y. L. Tissue cells feel and respond to the stiffness of their substrate. *Science* **310**, doi:10.1126/science.1116995 (2005). 10.1126/science.1116995 16293750

[15] Engler AJ (2004). Myotubes differentiate optimally on substrates with tissue-like stiffness: pathological implications for soft or stiff microenvironments. Journal of Cell Biology.

[16] Banwell EF (2009). Rational design and application of responsive alpha-helical peptide hydrogels. Nature Materials.

[17] Collier, J. H. Modular self-assembling biomaterials for directing cellular responses. *Soft Matter* **4**, doi:10.1039/b805563g (2008). 10.1039/b805563g PMC2829887 20198120

[18] Silva GA (2004). Selective differentiation of neural progenitor cells by high-epitope density nanofibers. Science.

[19] Webber MJ, Appel EA, Meijer EW, Langer R (2016). Supramolecular biomaterials. Nature Materials.

[20] Pashuck ET, Cui H, Stupp SI (2010). Tuning Supramolecular Rigidity of Peptide Fibers through Molecular Structure. Journal of the American Chemical Society.

[21] Discher DE, Mooney DJ, Zandstra PW (2009). Growth Factors, Matrices, and Forces Combine and Control Stem Cells. Science.

[22] Mei Y (2010). Combinatorial development of biomaterials for clonal growth of human pluripotent stem cells. Nature Materials.

[23] Dalby MJ (2007). The control of human mesenchymal cell differentiation using nanoscale symmetry and disorder. Nature Materials.

[24] Engler AJ, Sen S, Sweeney HL, Discher DE (2006). Matrix elasticity directs stem cell lineage specification. Cell.

[25] Alakpa EV (2016). Tuneable supramolecular hydrogels for selection of lineage guiding metabolites in stem cell cultures. Chem.

[26] Gilbert PM (2010). Substrate Elasticity Regulates Skeletal Muscle Stem Cell Self-Renewal in Culture. Science.

[27] Jayawarna V (2006). Nanostructured hydrogels for three-dimensional cell culture through self-assembly of fluorenylmethoxycarbonyl-dipeptides. Advanced Materials.

[28] Mahler A, Reches M, Rechter M, Cohen S, Gazit E (2006). Rigid, self-assembled hydrogel composed of a modified aromatic dipeptide. Advanced Materials.

[29] Jayawarna V (2009). Introducing chemical functionality in Fmoc-peptide gels for cell culture. Acta Biomaterialia.

[30] Nguyen, B. V. *et al*. Biomechanical properties of single chondrocytes and chondrons determined by micromanipulation and finite-element modelling. *Journal of the Royal Society Interface* **7**, doi:10.1098/rsif.2010.0207 (2010). 10.1098/rsif.2010.0207 PMC2988268 20519215

[31] Ofek, G. & Athanasiou, K. A. Micromechanical properties of chondrocytes and chondrons: Relevance to articular cartilage tissue engineering. *Journal of Mechanics of Materials and Structures* **2**, doi:10.2140/jomms.2007.2.1059 (2007).

[32] Caron MMJ (2012). Redifferentiation of dedifferentiated human articular chondrocytes: comparison of 2D and 3D cultures. Osteoarthritis and Cartilage.

[33] Darling EM (2009). Mechanical Properties and Gene Expression of Chondrocytes on Micropatterned Substrates Following Dedifferentiation in Monolayer. Cellular and Molecular Bioengineering.

[34] Matsumoto E, Furumatsu T, Kanazawa T, Tamura M, Ozaki T (2012). ROCK inhibitor prevents the dedifferentiation of human articular chondrocytes. Biochemical and Biophysical Research Communications.

[35] Maleski MP, Knudson CB (1996). Hyaluronan-mediated aggregation of limb bud mesenchyme and mesenchymal condensation during chondrogenesis. Experimental Cell Research.

[36] Tachetti C (1992). Cell condensation in chondrogenic differentiation. Experimental Cell Research.

[37] Bosnakovski D (2006). Chondrogenic differentiation of bovine bone marrow mesenchymal stem cells (MSCs) in different hydrogels: Influence of collagen type II extracellular matrix on MSC chondrogenesis. Biotechnology and Bioengineering.

[38] Coates EE, Riggin CN, Fisher JP (2013). Photocrosslinked alginate with hyaluronic acid hydrogels as vehicles for mesenchymal stem cell encapsulation and chondrogenesis. Journal of Biomedical Materials Research Part A.

[39] Ma K, Titan AL, Stafford M, Zheng CH, Levenston ME (2012). Variations in chondrogenesis of human bone marrow-derived mesenchymal stem cells in fibrin/alginate blended hydrogels. Acta Biomaterialia.

[40] Schuurman W (2009). Zonal Chondrocyte Subpopulations Reacquire Zone-Specific Characteristics During *In Vitro* Redifferentiation. American Journal of Sports Medicine.

[41] Watts AE, Ackerman-Yost JC, Nixon AJ (2013). A Comparison of Three-Dimensional Culture Systems to Evaluate *In Vitro* Chondrogenesis of Equine Bone Marrow-Derived Mesenchymal Stem Cells. Tissue Engineering Part A.

[42] Ekanayake S, Hall BK (1994). Hypertrophy is not a prerequisite for type-x collagen expression or mineralization of chondrocytes derived from cultured chick mandibular ectomesenchyme. International Journal of Developmental Biology.

[43] Gerstenfeld LC, Landis WJ (1991). Gene-expression and extracellular-matrix ultrastructure of a mineralizing chondrocyte cell-culture system. Journal of Cell Biology.

[44] Sullivan TA, Uschmann B, Hough R, Leboy PS (1994). Ascorbate modulation of chondrocyte gene-expression is independent of its role in collagen secretion. Journal of Biological Chemistry.

[45] Childs AC, Mehta DJ, Gerner EW (2003). Polyamine-dependent gene expression. Cellular and Molecular Life Sciences.

[46] Igarashi K, Kashiwagi K (2010). Modulation of cellular function by polyamines. The international journal of biochemistry & cell biology.

[47] Facchini A (2012). Role of polyamines in hypertrophy and terminal differentiation of osteoarthritic chondrocytes. Amino Acids.

[48] Mwale, F., Stachura, D., Roughley, P. & Antoniou, J. Limitations of using aggrecan and type X collagen as markers of chondrogenesis in mesenchymal stem cell differentiation. *Journal of Orthopaedic Research* **24**, doi:10.1002/jor.20200 (2006). 10.1002/jor.20200 16779832

[49] Hwang WS (1992). Collagen fibril structure of normal, aging, and osteoarthritic cartilage. Journal of Pathology.

[50] Wong, M. & Carter, D. R. Articular cartilage functional histomorphology and mechanobiology: a research perspective. *Bone* **33**, doi:10.1016/s8756-3282(03)00083-8 (2003). 10.1016/s8756-3282(03)00083-8 12919695

[51] Schefe, J. H., Lehmann, K. E., Buschmann, I. R., Unger, T. & Funke-Kaiser, H. Quantitative real-time RT-PCR data analysis: current concepts and the novel “gene expression’s C-T difference” formula. *Journal of Molecular Medicine-Jmm* **84**, doi:10.1007/s00109-006-0097-6 (2006). 10.1007/s00109-006-0097-6 16972087

[52] Creek, D. J., Jankevics, A., Burgess, K. E. V., Breitling, R. & Barrett, M. P. IDEOM: An Excel interface for analysis of LC-MS based metabolomics data. *Bioinformatics*, doi:10.1093/bioinformatics/bts069 (2012). 10.1093/bioinformatics/bts069 22308147

[53] Scheltema RA, Jankevics A, Jansen RC, Swertz MA, Breitling R (2011). PeakML/mzMatch: A File Format, Java Library, R Library, and Tool-Chain for Mass Spectrometry Data Analysis. Analytical Chemistry.

[54] Compton, S. J. & Jones, C. G. Mechanism of dye response and interference in the bradford protein assay. *Analytical Biochemistry* **151**, doi:10.1016/0003-2697(85)90190-3 (1985). 10.1016/0003-2697(85)90190-3 4096375

[55] Creek DJ (2011). Toward Global Metabolomics Analysis with Hydrophilic Interaction Liquid Chromatography-Mass Spectrometry: Improved Metabolite Identification by Retention Time Prediction. Analytical Chemistry.

[56] Xia J, Psychogios N, Young N, Wishart DS (2009). MetaboAnalyst: a web server for metabolomic data analysis and interpretation. Nucleic Acids Research.

