# Supplementary material for: Improving cartilage phenotype from differentiated pericytes in tunable peptide hydrogels
Source: Sci Rep. 2017 Jul 31;7:6895. doi: 10.1038/s41598-017-07255-z (PMC5537289; doi:10.1038/s41598-017-07255-z)

Supplementary Information

Improving cartilage phenotype from differentiated pericytes in tunable peptide hydrogels

Enateri V. Alakpa [1*], Vineetha Jayawarna [2], Karl E. V. Burgess [3], Christopher C. West [4], Bruno Péault [4, 5], Rein V. Ulijn [6] & Matthew J. Dalby [2].

1. Institution for Integrative Medical Biology. Umeå University, SE901 87 Umeå, Sweden
2. Centre for Cell Engineering. Institute of Molecular, Cell & Systems Biology. College of Medical, Veterinary & Life Sciences. Joseph Black Building, University of Glasgow. Glasgow G12 8QQ, UK.
3. Scottish Polyomics Facility. Wolfson Wohl Cancer Research Centre, College of Medical, Veterinary & Life Sciences, University of Glasgow, Garscube Estate, Glasgow G61 1QH, UK.
4. Centre for Regenerative Medicine and Centre for Cardiovascular Science, University of Edinburgh, Edinburgh EH16 4UU, UK.
5. Orthopaedic Surgery Dept and Broad Stem Cell Research Center, University of California, Los Angeles, USA.
6. Advanced Science Research Center (ASRC), City University of New York, New York, NY 10031, USA.

*Address correspondence to: Enateri.Alakpa@umu.se

Supplementary Table S1: Demographic data and cell metrics from the 3 donors used in this study.

Donor	Sex	Age	Total nucleated cells in SVF per 100cc of adipose tissue	% viability	% pericytes
1	M	35	50×10^6	72.8	7.3
2	F	44	48×10^6	81.3	8.5
3	F	50	52×10^6	92.8	11.5

Supplementary Figure S1: Demonstration of purity of pericyte cultures using flow cytometry. Passage 3 pericytes demonstrating uniform positive expression of CD146 (>99.99%), and negative expression of CD31 and CD45 versus isotype control. CD146-Alexa647 (1:100, AbD Serotec, Raleigh, NC), CD45 APC-cy7 and CD31-FITC (1:100, all from BD Biosciences, San Jose, CA).

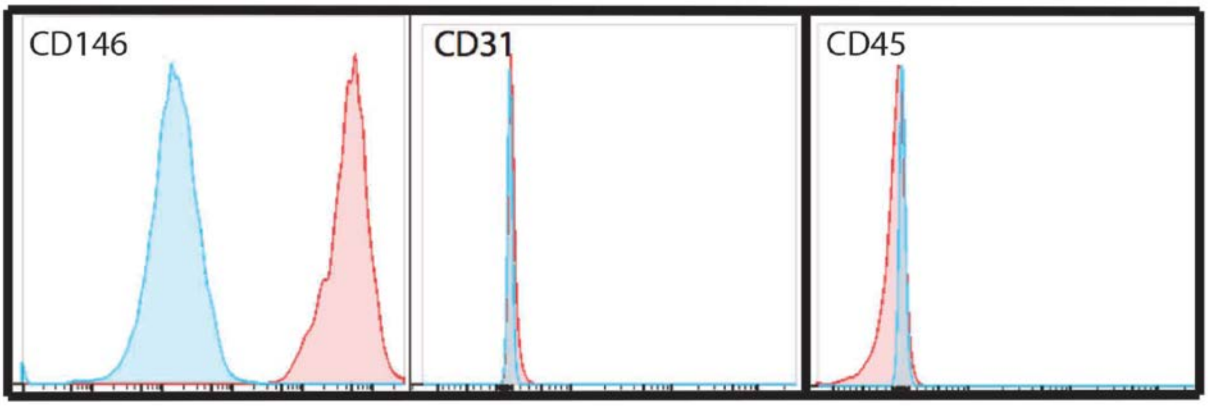

Supplementary Figure S2: Confocal microscopy images of F_2/S hydrogels immunostained for either aggrecan or type II collagen. Pericyte cells were cultured for 28 days within the F_2/S hydrogels prior to staining. Images were obtain from hydrogels which contained no cells (control, F_2/S), hydrogels which contained cells and cultured in basal media (F_2/S -) and chondrogenic induction media (F_2/S +).

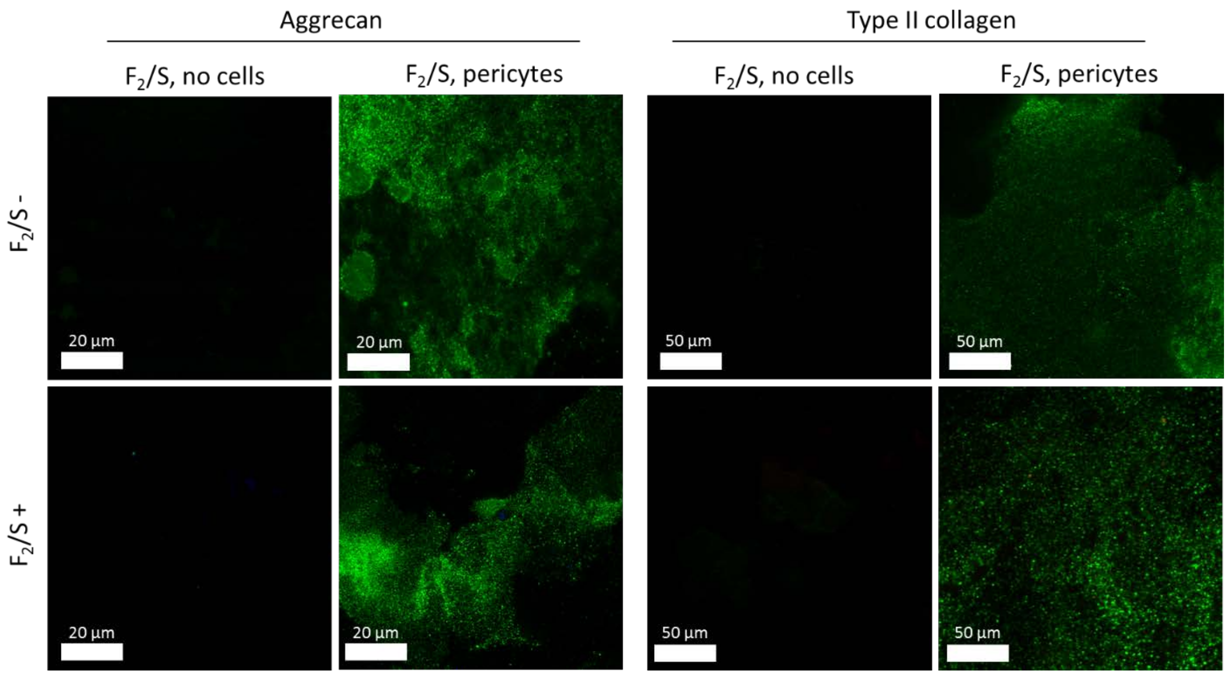

Supplement: Supplementary file 1 — Supplementary information [file 41598_2017_7255_MOESM1_ESM.pdf]
